# Walker occupancy has an impact on changing airborne bacterial communities in an underground pedestrian space, as small-dust particles increased with raising both temperature and humidity

**DOI:** 10.1371/journal.pone.0184980

**Published:** 2017-09-18

**Authors:** Torahiko Okubo, Takako Osaki, Eriko Nozaki, Akira Uemura, Kouhei Sakai, Mizue Matushita, Junji Matsuo, Shinji Nakamura, Shigeru Kamiya, Hiroyuki Yamaguchi

**Affiliations:** 1 Department of Medical Laboratory Science, Faculty of Health Sciences, Hokkaido University, Kita-ku, Sapporo, Japan; 2 Department of Infectious Diseases, Kyorin University School of Medicine, Shinkawa, Mitaka, Tokyo, Japan; 3 Division of Biomedical Imaging Research, Juntendo University Graduate School of Medicine, Bunkyo-ku, Tokyo, Japan; Ecole des Mines d'Ales, FRANCE

## Abstract

Although human occupancy is a source of airborne bacteria, the role of walkers on bacterial communities in built environments is poorly understood. Therefore, we visualized the impact of walker occupancy combined with other factors (temperature, humidity, atmospheric pressure, dust particles) on airborne bacterial features in the Sapporo underground pedestrian space in Sapporo, Japan. Air samples (*n* = 18; 4,800L/each sample) were collected at 8:00 h to 20:00 h on 3 days (regular sampling) and at early morning / late night (5:50 h to 7:50 h / 22:15 h to 24:45 h) on a day (baseline sampling), and the number of CFUs (colony forming units) OTUs (operational taxonomic units) and other factors were determined. The results revealed that temperature, humidity, and atmospheric pressure changed with weather. The number of walkers increased greatly in the morning and evening on each regular sampling day, although total walker numbers did not differ significantly among regular sampling days. A slight increase in small dust particles (0.3–0.5μm) was observed on the days with higher temperature regardless of regular or baseline sampling. At the period on regular sampling, CFU levels varied irregularly among days, and the OTUs of 22-phylum types were observed, with the majority being from *Firmicutes* or *Proteobacteria* (γ-), including *Staphylococcus* sp. derived from human individuals. The data obtained from regular samplings reveled that although no direct interaction of walker occupancy and airborne CFU and OTU features was observed upon Pearson's correlation analysis, cluster analysis indicated an obvious lineage consisting of walker occupancy, CFU numbers, OTU types, small dust particles, and seasonal factors (including temperature and humidity). Meanwhile, at the period on baseline sampling both walker and CFU numbers were similarly minimal. Taken together, the results revealed a positive correlation of walker occupancy with airborne bacteria that increased with increases in temperature and humidity in the presence of airborne small particles. Moreover, the results indicated that small dust particles at high temperature and humidity may be a crucial factor responsible for stabilizing the bacteria released from walkers in built environments. The findings presented herein advance our knowledge and understanding of the relationship between humans and bacterial communities in built environments, and will help improve public health in urban communities.

## Introduction

Humans spends over 90% of their time in indoor environments such as houses, offices, schools, hospitals, restaurants, and other public spaces such as underground pedestrian walkways [1–3]; therefore, these environments should be maintained in a clean state without air pollution. Furthermore, the large number of bacteria emitted from the skin and oral cavities of humans through coughing, sneezing, talking, and breathing indicates that human occupancy is a source of indoor airborne bacteria [4–7]. Therefore, air pollution of indoor environments with human pathogens can hinder individual health by spreading or exacerbating disease [8–10]. Accordingly, microbial communities have been actively monitored in distinct indoor public environments to understand their dynamics [11].

The features and rate of bacterial shedding from individuals could be reflected in the bacterial community structures of built environments; therefore, they may serve as markers for estimating healthy indoor air conditions. Although building ventilation systems and sanitation can influence the community structure of indoor air bacteria, the abundance of human-derived bacteria is more than two-fold greater in human occupied indoor environments than unoccupied areas [12–14]. Sweeping of floors and dusting of objects also likely influence bacterial communities through the suspension of bacteria released from individuals in the built environment. Indeed, *Streptococcus* and *Staphylococcus*, which are generally found in human normal flora, are frequently detected in the air of healthy-office building environments [15–18]. Although it is clear that human occupancy influences bacterial communities in indoor air, it is not known whether the movement of individuals (e.g., walking) has an impact on these communities.

Air also contains a significant number of dust particles composed of both inorganic and organic materials, including bacteria, and most airborne bacteria found indoors are on particles with diameters of 0.1–10 μm [19, 20]. Accordingly, it is possible that resuspension of settled dust particles by walking or other movements can influence bacterial communities [21–23]. It is also feasible that changing temperature, humidity or atmospheric pressure could influence the movement of dust particles with bacteria, presumably facilitating their circulation into built environments [24–26]. Thus, changes in indoor bacterial communities in built environments are very dynamic and complicated. However, it is not clear how such factors could influence the features of bacteria suspended by or released from walkers in actual indoor environments, such as public underground pedestrian spaces.

Therefore, we visualized impact of walker occupancy with other factors including temperature, humidity, atmospheric pressure, and dust particle numbers on changing airborne bacterial features in a built environment, the Sapporo underground pedestrian space, located in Sapporo, Japan. The results revealed an impact of walker occupancy on airborne bacteria that was influenced by temperature and humidity.

## Results

### Sapporo underground pedestrian space and limitation on air sampling

**Fig 1:**
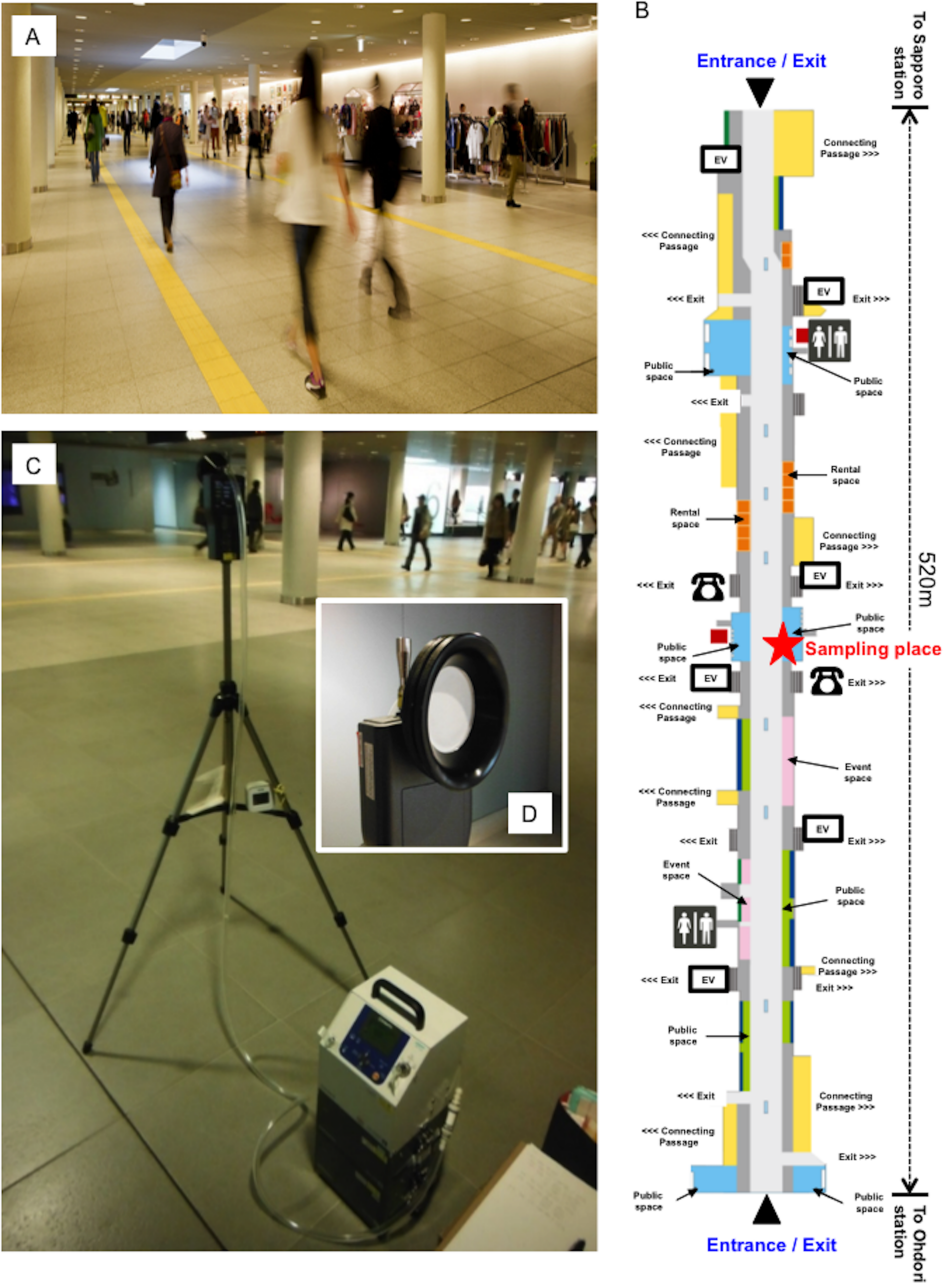
Sapporo underground pedestrian space and air sampling locations. (A) Representative features showing the pedestrian with walkers. (B) Map showing the pedestrian structure with the location of public spaces or entrances/exits. The photo and the map were obtained with permission from the following website (http://www.sapporo-chikamichi.jp/). (C) Image taken during air sampling. As mentioned in the text, the sampler was approximately 3 m from the wall. (D) Air sampler filter opening. The opening was adjusted to be 1.5 m from the floor to align with pedestrian head. The filter consisting of PTFE-binding glass fiber filter was pointed toward the center of the pedestrian passageway.

The study space was an underground pedestrian passageway 520 m in length and 16–18 m in width that was built in 2011. There is open access to the area from 5:45 h to 0:30 h throughout the year (Fig 1A and 1B). The pedestrian walkway connects two subway stations between Sapporo and Odori and is used by almost 50,000 people per day (City of Sapporo: http://www.city.sapporo.jp/city/english/index.html). Because it is a public facility, locations and access times for air sampling were restricted. Therefore, locations and times were selected based on guidance from the City of Sapporo, which maintains the pedestrian space. Accordingly, samples were collected from the center of the walkway (Fig 1B, red star marked on the map) on May 2, June 1, and July 5 (8:00 h to 20:00 h) (regular sampling), and July 15 (5:50 h to 7:50 h / 22:15 h to 24:45 h) (baseline sampling), which correspond to the larger temperature changes in a year, an optimum period for assessing the effect of temperature or humidity on changing the amount of dust particles or bacteria with bacterial flora dynamics at the pedestrian space. To prevent hindrance of pedestrians, the samples were collected approximately 3 m from the wall at 1.5 m from the floor (to approximate the height of the pedestrians), according to a previous report [27]. Samples were collected from a device (an air sampler with PTFE-binding glass fiber filter) that was aimed toward the center of the walkway (Fig 1C and 1D). In addition, since it was expected few pedestrian flow at both early morning and late night showing the baseline of dynamic changes at the space, we therefore performed air sampling at both early (5:50 h to 7:50 h) and late night (22:15 h to 24:30 h) on July 15 as a control.

### Changes in pedestrian numbers and environmental factors (weather, temperature, humidity, atmospheric pressure, and dust particle numbers) in the underground pedestrian space

**Fig 2:**
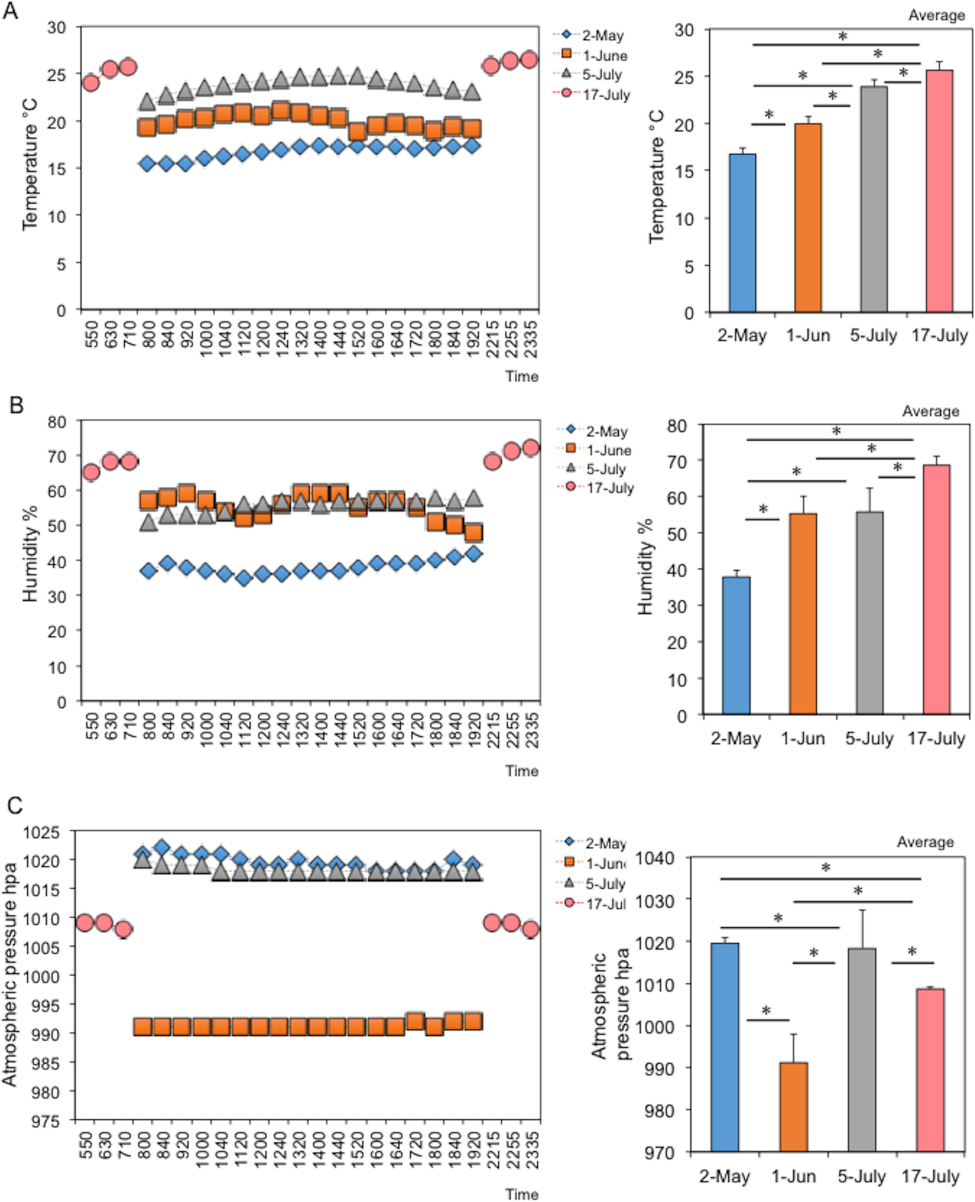
Changes in environmental factors (temperature, humidity and atmospheric pressure) in the underground pedestrian space. (A) Changes in temperature (°C). (B) Changes in humidity (%). (C) Changes in atmospheric pressure (hpa). Left and right panels show the daily and monthly changes, respectively. Asterisks indicate a significant difference (*p*<0.05) between bars connected by lines.

The weather differed on different sampling days (Japan Weather Association: http://www.tenki.jp/), being cloudy (rainfall: 0.0 mm) on May 2, slightly rainy (rainfall: 0.5 mm) on June 1, fine (rainfall: 0.0 mm) on July 5, and fine (rainfall: 0.0 mm) on July 15. Temperature, humidity, and atmospheric pressure were measured at 18 time points from 8:00 h to 20:00 h on each regular sampling day (May 2, June 1, July5) or at 6 time points from 5:50 h to 7:50 h and 22:15 h to 24:45 h (July 15) on baseline sampling day. As shown in Fig 2, the average temperature, humidity, and atmospheric pressure were 16.7±0.7°C, 37.9±1.8%, and 1,019±1.2 hPa on May 2, 19.9±0.7°C, 55.3±4.8%, and 991±6.8 hPa on June 1, and 23.8±0.7°C, 55.7±6.6%, 1,018±9.2 hPa on July 5, and 25.7±0.9°C, 68.7±2.5%, and 1,008±0.5 hPa on July 15, respectively. Both the average temperature and humidity were significantly changed depending on sampling day (Fig 2, upper- and middle-right figures), although the changes in both factors were minimal during each of the days (Fig 2, upper- and middle-left figures). However, the average atmospheric pressure was significantly lower on June 1 than on the other days (Fig 2, lower figures). Thus, although temperature, humidity, and atmospheric pressure on each of the days varied, these variations were minimal during each sampling day because the study area was underground.

**Fig 3:**
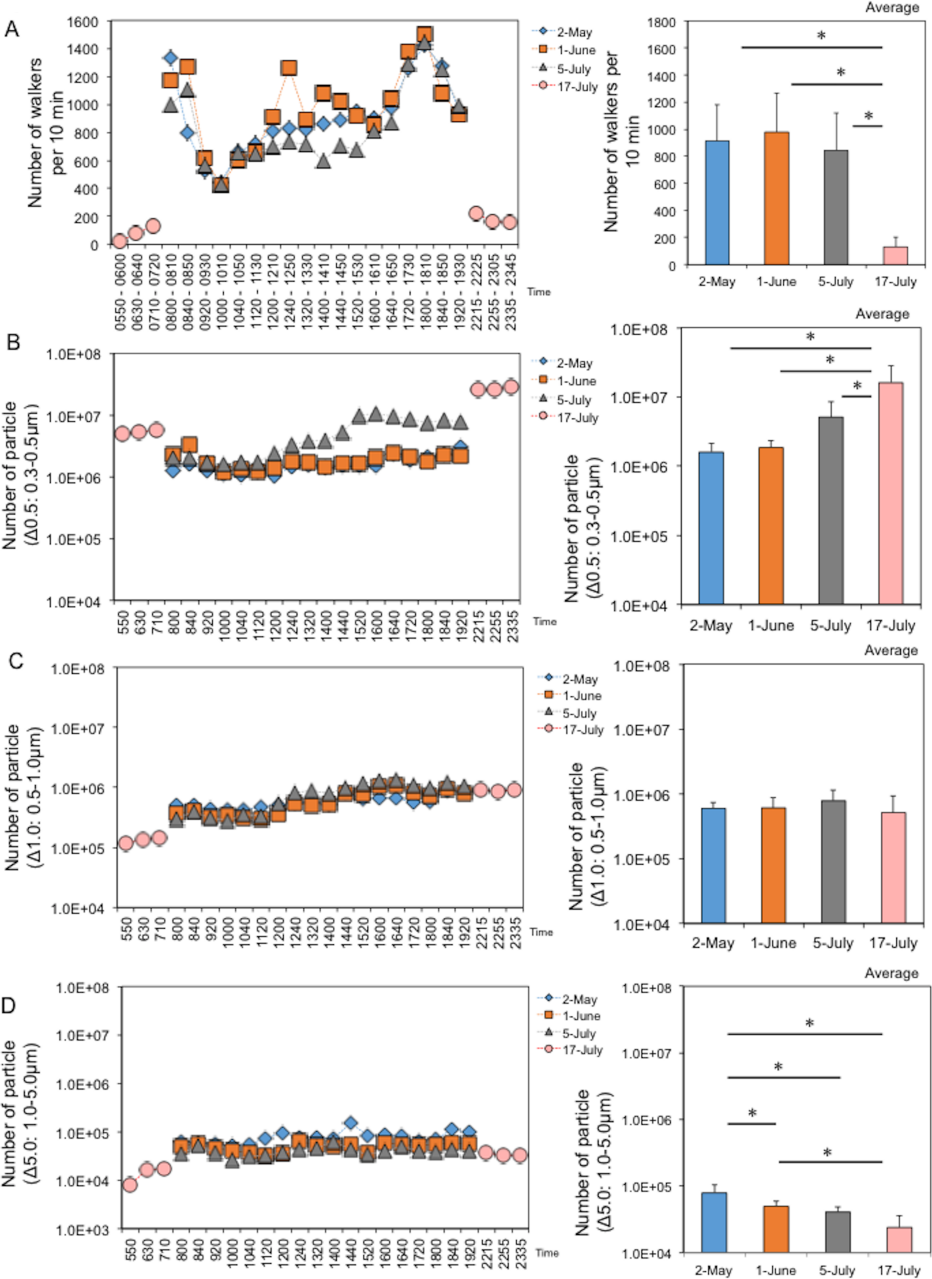
Changes in walker numbers and environmental factors (dust particle numbers) in the underground pedestrian space. (A) Changes in walker numbers. (B) Changes in particle numbers (particle size Δ0.5: 0.3–0.5μm). (C) Changes in numbers of particles (particle size Δ1.0: 0.5–1.0μm). (D) Changes in numbers of particles (particle size Δ5.0: 1.0–5.0μm). Left and right panels show daily and monthly changes, respectively. Asterisks indicate a significant difference (*p*<0.05) between bars connected by lines.

Pedestrian and particle numbers were also measured at 18 time points from 8:00 h to 20:00 h on each regular sampling day, and at 6 time points from 5:50 h to 7:50 h and 22:15 h to 24:35 h on baseline sampling day. When compared among regular sampling days, there was no significant difference in the average number of walkers per 10 min among sampling days (Fig 3A, right panel). Because of the rush hours, the number of walkers increased dramatically from 8:00–8:50 h and 17:20–18:10. The variations in pedestrian numbers were very similar among sampling days (Fig 3A, left panel), indicating that the pedestrian space was regularly used for commuting, with few people gathering for events. Meanwhile, as expected, the number of walkers was minimal at early morning and late night, indicating an appropriate period for determining baseline with few walkers (Fig 3A). The number of floating dust particles in the pedestrian space was also determined automatically by a dust particle counter (See Fig 1D, protruded stainless tube attached into the back of the air filter). In contrast to the number of walkers, the particle numbers remained relatively stable throughout the day, regardless of particle size (Fig 3B, 3D and 3D, left panels). The average number of dust particles measured at each time point was 1,610,762 (Δ0.5), 59,461(Δ1.0), and 79,213 (Δ5.0) on May 2, 1,848,919 (Δ0.5), 607,355 (Δ1.0), and 49,743 (Δ5.0) on June 1, 5,115,582 (Δ0.5), 780,465 (Δ1.0), and 40,939 (Δ5.0) on July 5, and 16,026,080 (Δ0.5), 511,554 (Δ1.0), and 24,042 (Δ5.0) on July 15, respectively (Fig 3B, 3C and 3D, right panels). Meanwhile, the number of Δ0.5 particles differed significantly among sample dates, indicating that changes with increasing temperature could positively influence the amount of small floating dust particles, although assessed time points were limited. No change in Δ1.0 was observed. Also, the number of Δ5.0 decreased significantly with increasing temperature. Taken together, the results showed a complicated behavior of floating dust particles that varied with temperature and humidity depending on particle size.

### Changes in CFU numbers and phylum types on the underground pedestrian space

**Fig 4:**
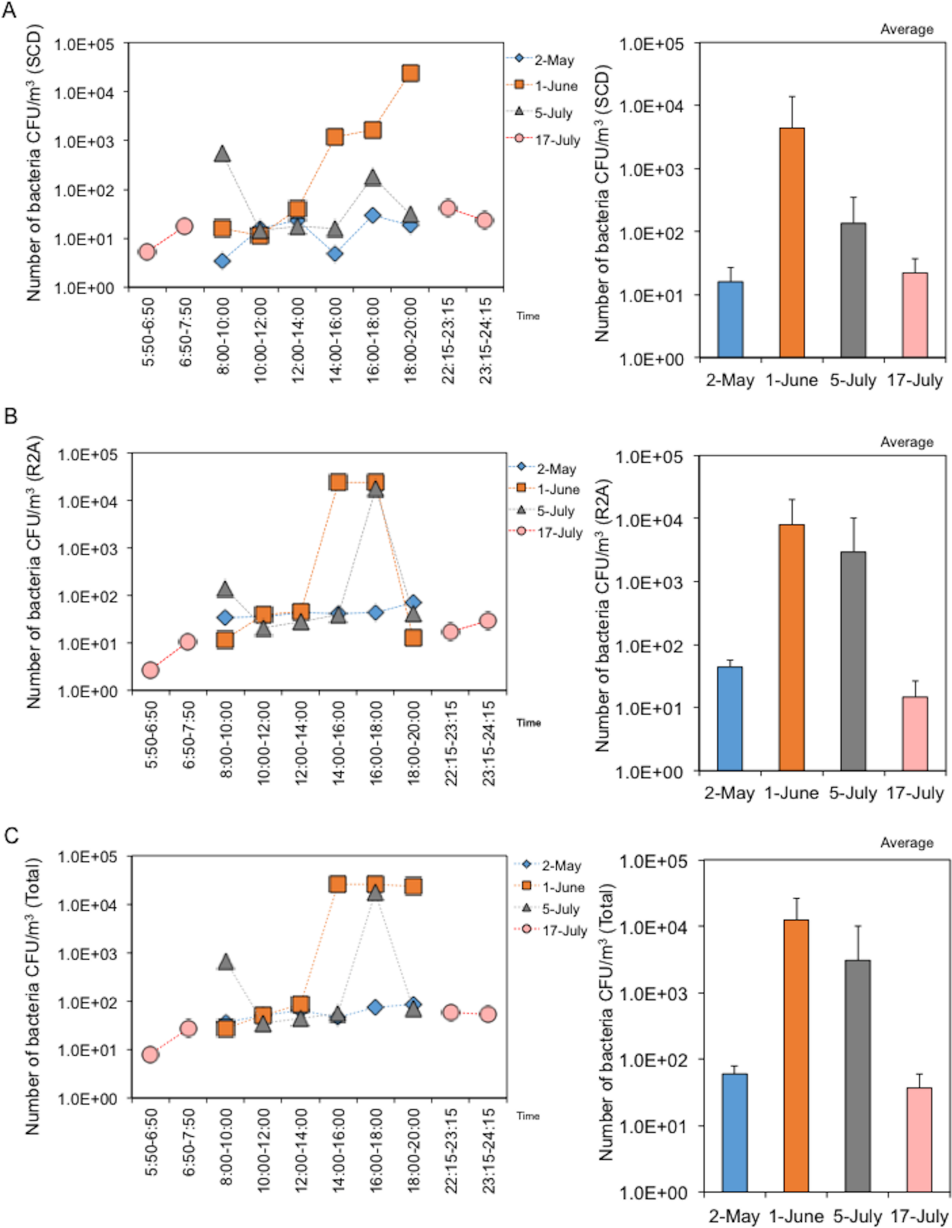
Changes in CFU numbers in underground pedestrian space. (A) Changes in CFU numbers estimated by the SCD plate culture (rich medium for “walkers”). (B) Changes in CFU numbers estimated by R2A plate culture (poor medium for “built environment”). (C) Total CFUs. Left and right panels show daily and monthly changes, respectively.

**Fig 5:**
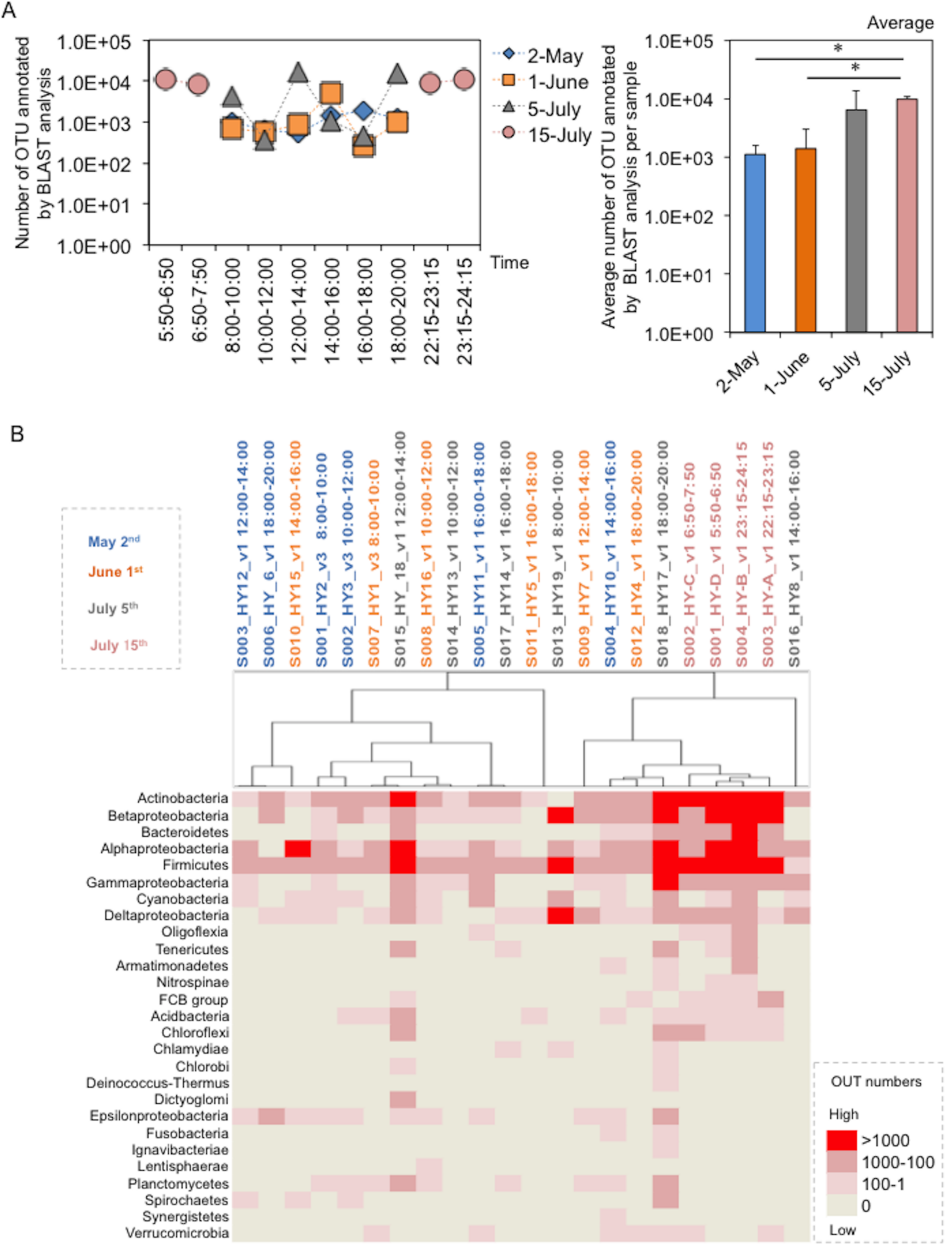
Changes in OTU numbers and phylum types on the underground pedestrian space. (A) Changes in OTU numbers estimated based on 16S rDNA sequence analysis and BLAST search. Left and right panels show daily and monthly changes, respectively. (B) Clustering of the phylum type defined by 16SrDNA sequencing. As mentioned in the text, the OTUs obtained from the mock control were removed from those obtained for each sample. These OTU data were similarly processed with a setting [>80% filtering and Pearson correlation (centered)]. After replaced to the files with a ‘cdt’ extension in the software of Cluster 3.0 for visualizing on the TreeViewX.

A total of 4,800 L of air was trapped on a filter every 2 hours on each regular sampling day from 8:00–20:00 h or every 1 hours on each baseline sampling day from 5:50 to 7:20 h and 22:15 to 24:15 h. Each of the filter-rinsed solution samples was divided into two tubes to determine the number of CFUs and for DNA extraction followed by 16S rDNA sequencing. Because the airborne bacteria originated from the built environments and the pedestrians themselves, CFUs were enumerated by culture on SCD (soybean-casein digested) (nutrient rich medium to reflect pedestrians) and R2A plates (nutrient poor medium to reflect the built environment). The average number of CFUs was 59.9±19 (May 2), 12,366±13,507 (June 1), 3,069±7,058 (July 5), and 36.8±23.4 (July 15). In contrast to the environmental factors shown above, there were no significant differences in CFUs observed among sampling days, although there appeared to be complicatedly but a change depending on up/down of temperature and humidity with walker numbers (Fig 4, right-lower panels). Moreover, the CFUs varied with no specific pattern within each sampling date, although they tended to increase in the evening on all days except May 2 (Fig 4, left-lower panels). There were also no obvious differences in the number of CFUs observed in the SCD and R2A cultures (Fig 4, left-upper and -meddle panels). These findings indicated that airborne bacteria shaping bacterial features in the space were accidentally changed. Furthermore, it was notable that the number of CFUs (both SCD and R2A) was minimal at early and late night times with few walkers. We also determined the number of OTUs with phylum type of bacteria by 16S rDNA sequencing and BLAST analysis. The average OTU number estimated by sequencing was 1,110±485 (May 2), 1,392±1,737 (June 1), 6,413±7,606 (July 5), and 9,752±1,465 (July 5), indicating a slight increase in OTU numbers with time (Fig 5A). Moreover, 22-phylum types were identified, with the majority being *Firmicutes* or *Proteobacteria* (γ-) (including *Staphylococcus*); *Proteobacteria* is distinctively shown into 5 classes (α-ε). Interestingly, cluster analysis revealed that the numbers of OTUs increased depending on time changes with increases in temperature and humidity (Fig 5B).

### Variations in airborne bacterial communities in an underground pedestrian space in conjunction with increases in small-dust particles and increasing temperature and humidity

**Fig 6:**
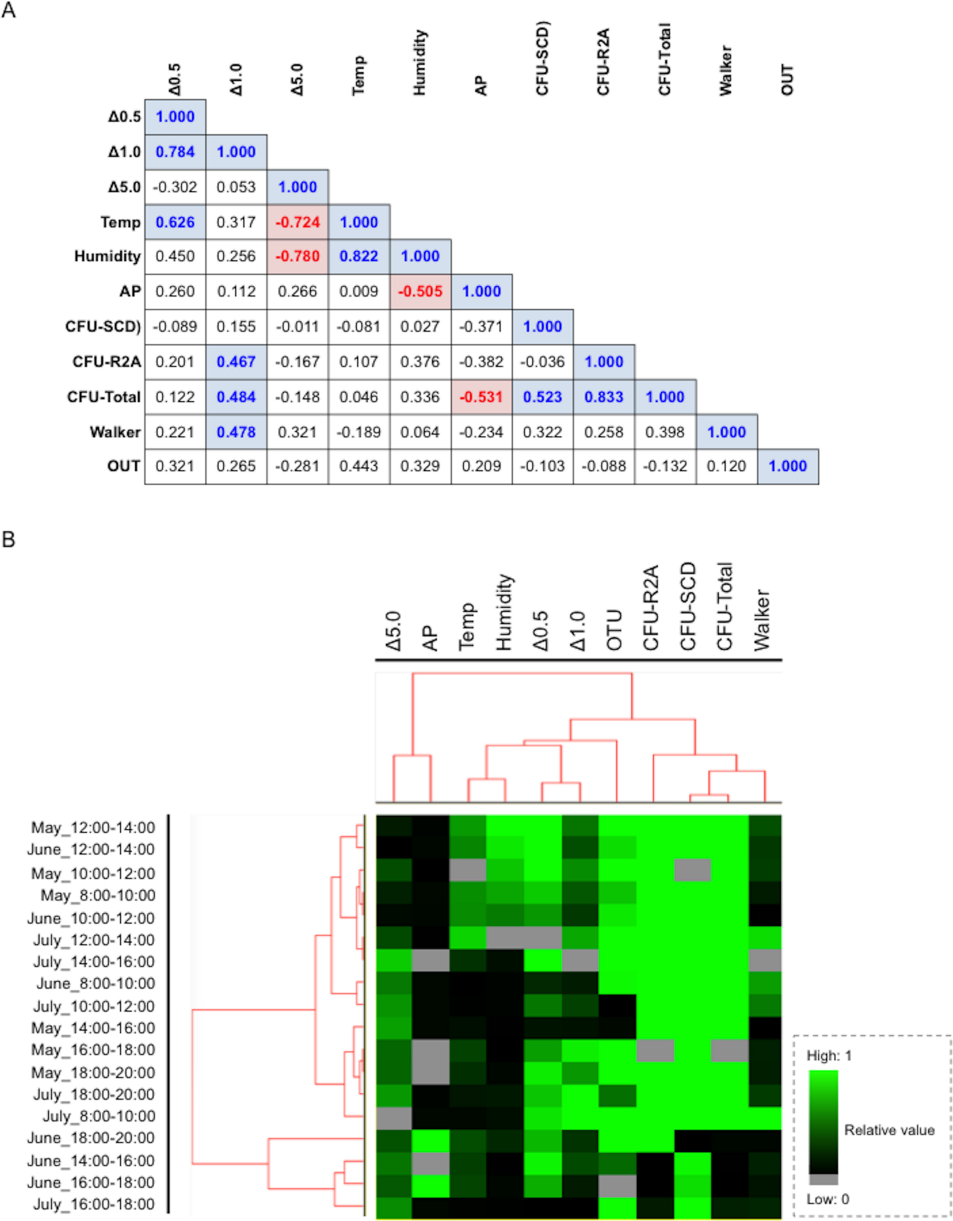
Comparison of Pearson's correlation coefficient among factors and phylum types in the underground pedestrian space. (A) Matrix showing comparison of Pearson's correlation coefficient among factors. Values show the correlation coefficient. Bold values showing positive and negative correlation with colors indicate statistical significance (a correlation coefficient value of >0.5 or <−0.05 with a *p*-value < 0.05). (B) Clustering of the phylum type consisting of distinct factors. All values of the factors with distinct ranges were adjusted to an equivalent range from “0” to “1”. Factors include Δ0.5 (particle size: 0.3–0.5μm), Δ1.0 (particle size: 0.5–1.0μm), Δ5.0 (particle size: 1.0–5.0μm), Temp (temperature: °C), Humidity (%), AP (atmospheric pressures: hpa), CFU-SCD (CFU numbers per m^3^ estimated by SCD plate culture), CFU-R2A (CFU numbers per m^3^ estimated by R2A plate culture), CFU-Total (total CFU numbers per m^3^ estimated by SCD and R2A plate cultures), Walker (walker numbers per 10 min) and OTU (OTU numbers estimated by 16S rDNA sequence analysis and BLAST search). These data were similarly processed with a setting (>80% filtering and Spearman rank correlation). After replaced to the files with a ‘cdt’ extension in the software of Cluster 3.0 for visualizing on the TreeViewX.

Pearson’s correlation analysis and cluster analysis were conducted to evaluate the relationship between airborne bacteria and the degree of walker occupancy, as well as changes in environmental factors (temperature, humidity) and the amount of dust particles. A significant positive correlation (correlation coefficient: >0.4 with *p*<0.05) was observed for the following combinations: Δ0.5 and Δ1.0, Δ0.5 and temperature, Δ1.0 and CFU (total), Δ1.0 and walker, and temperature and humidity (Fig 6A, blue bars). Conversely, Δ5.0 and temperature, Δ5.0 and humidity, and atmospheric pressure and CFUs (total) were negatively correlated (Fig 6A, red bars). The cluster analysis revealed two clusters, with an independent small group consisting of atmospheric pressure and Δ5.0, and a large group consisting of temperature, humidity, Δ0.5, Δ1.0, CFU (R2A), CFU (SCD), CFU (total) and walkers, in order (Fig 6B), indicating an indirect interaction between walker occupancy and floating bacteria present in the public space. Overall, the results revealed that bacterial features could be drastically changed depending on walker occupancy, but only when increases of both temperature and humidity occurred in the presence of small dust particles.

## Discussion

Because a large number of bacteria are emitted from skin or oral cavities through coughing, sneezing, talking, and breathing, bacteria shed by humans are considered a source of airborne bacteria in built environments [4–11]. Moreover, most indoor air contains inorganic dust particles ranging from 0.1 to 10 μm in size [19, 20], which presumably serve as a platform for survival or transfer of floating bacteria. Furthermore, it is feasible that circulation of floating bacteria released from walkers into indoor environments could be easily influenced by changing environmental factors such as temperature or humidity [21–26]. However, the synergistic effects of walker occupancy and these environmental factors on bacterial community structures of actual built environments remains unknown. Therefore, we visualized the impact of walker occupancy with these factors on changing airborne bacterial communities in the Sapporo underground pedestrian space in Sapporo, Japan. Here, we demonstrate for the first time that floating bacteria in the environment could be altered by the degree of walker occupancy, with increases occurring in response to increasing temperature and humidity in the presence of small dust particles.

Variations in walker numbers were similar among sampling days, with peaks occurring in the morning and evening, although there was no difference in the average number of walkers between sampling days. These results indicate that the pedestrian space is regularly used for commuting to school or work. Although a small peak was observed around noon on June 1, this was because people entered the walkway to avoid a short period of rain. In contrast to the number of walkers, minimal changes in particle numbers were observed throughout the day. Although it is common for indoor air particles to be critically influenced by outdoor air [28–31], underground spaces with many passageways, but no doors leading outside, appear to be an exception. Also, because the total number of bacterial viable counts was approximately 10^3−4^ CFU, it is likely that most of the remaining particles were associated with inorganic dust that entered or resuspended the underground space with a minimal change. The changes of temperature and humidity also positively influenced the amount of airborne dust particles of small size. It is likely that the increasing temperature and humidity facilitated floating dust via increases in buoyancy. It is also feasible that large dust particles could become less buoyancy with increased weight. Recent studies showing a critical correlation of bacterial abundance with particle size in dust [32–34] support our hypothesis that small particles (less than 1.0 μm in size) could become a platform for bacteria, facilitating their circulation or survival in built environments. Thus, we found that the behavior of small particles was opposite to that of large particles in response to temperature and moisture changes. Since many studies of indoor microbiology have focused on bioaerosols present in floating dust particles [11, 20], these data provide us with valuable information for understanding the circulation of bacteria in indoor environments.

There was no significant difference in CFU numbers among sampling days. Moreover, the numbers of CFUs showed irregular variations throughout the day. Evaluation of OTUs revealed 22 phylum types, with the majority belonging to *Firmicutes* or *Proteobacteria* (γ-) (including *Staphylococcus*). Bacteria derived from human skin such as *Staphyloccocus* were also frequently isolated from air sampling solutions (data not shown). Meanwhile, cluster analysis revealed that the numbers of OTUs were influenced by the changes of temperature and humidity. As mentioned above, it is well known that a large number of bacteria are emitted from skin or oral cavities through coughing, sneezing, talking, and breathing [19–26]. Accordingly, it is feasible that shedding bacteria from walking and static individuals occurs in indoor built environments [35]. Thus, both moving and static humans influence the indoor bacterial community structure in the studied underground space.

**Fig 7:**
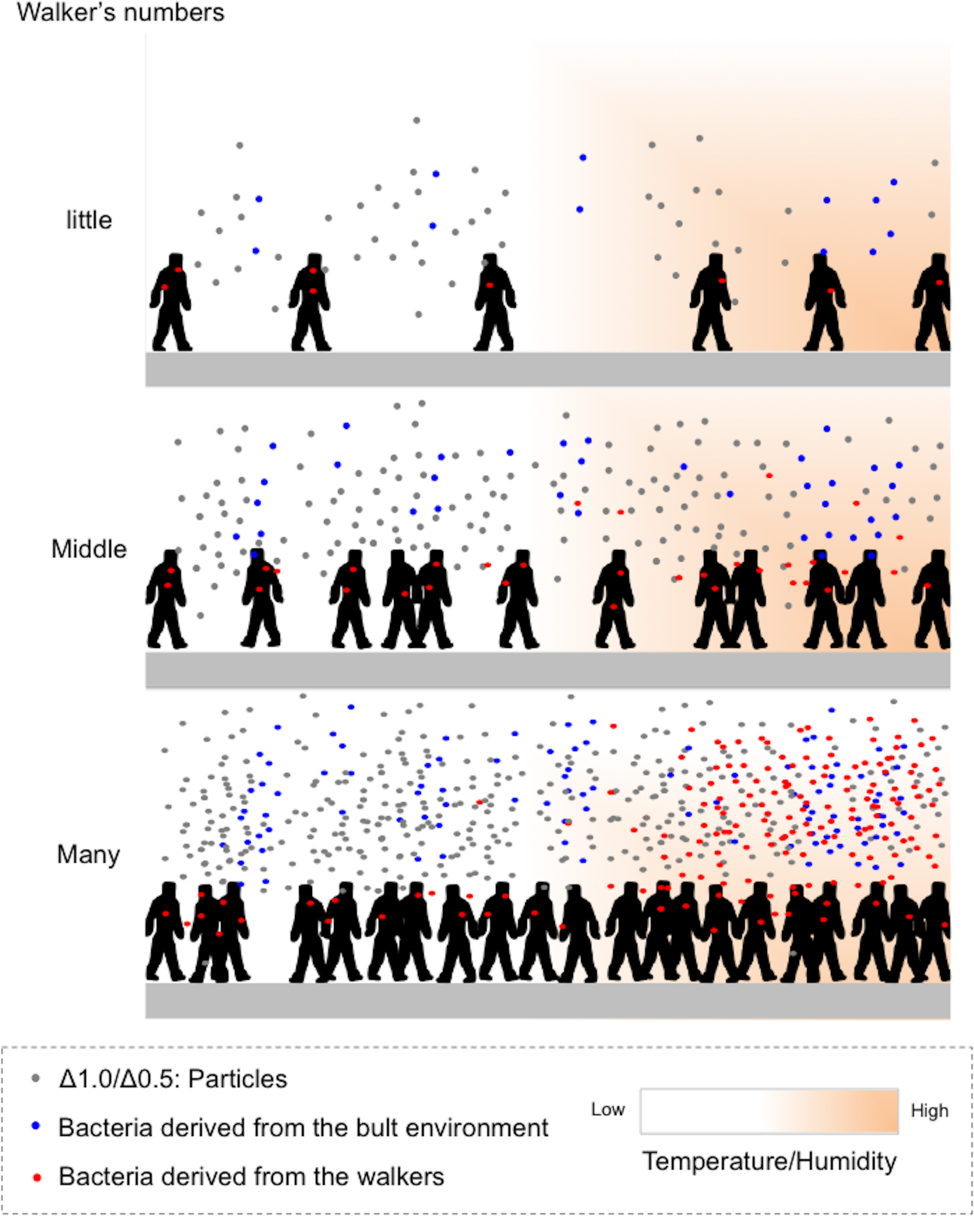
A possible scenario showing bacterial features released from walkers and the built environment. Upper panel (small number of walkers) showing the number of bacteria released from individuals is minimal. Middle panel (moderate number of walkers) showing that although increasing walker occupancy facilitates the number of floating small dust particles with bacteria released from individuals, the number of bacteria is still minimal. Lower panel (high number of walkers) showing that increasing temperature and humidity in the presence of a high concentration of small dust particles causes the bacteria released from walkers to reach the maximum level.

No direct interaction of walker occupancy with airborne CFUs and OTU features was seen upon analysis by Pearson's correlation coefficient test. However, cluster analysis indicated an obvious lineage consisting of walker occupancy, CFU numbers, OTU types, dust particles with small size, and seasonal factors, including temperature and humidity. These results suggest that walker occupancy could be indirectly related to airborne bacterial features in the underground space. We proposed the following possible scenario to explain the changing bacterial features in response to walkers (Fig 7). When there are few walkers in the space, the number of bacteria released from individuals is minimal (Fig 7, Upper panel ‘little’). Additionally, although increasing walker occupancy facilitates the number of floating small dust particles with bacteria released from individuals, the number of bacteria is still minimal (Fig 7, Middle panel ‘Middle’). However, the changes of temperature and humidity cause the number of bacteria released from walkers to reach their maximum levels in the presence of high concentrations of small dust particles (Fig 7, Lower panel ‘Many’). Thus, although additional studies should be conducted to clarify our results, our findings highlight the complicated interactive relationship between walker occupancy, increases in both temperature and humidity and the presence of a high number of small dust particles that could have an impact on airborne bacterial features in underground constructed spaces. The information provided in this study will facilitate improvement of public health in urban environments.

## Conclusions

Taken together, the results of this study indicate that there is a chain of positive correlations of walker occupancy with airborne bacteria in response to increased temperature and humidity in the presence of airborne small particles in the Sapporo underground pedestrian space. Thus, the small airborne particles of dust at high temperature and humidity may be a crucial factor facilitating the release of bacteria from walkers in constructed environments. These findings advance our knowledge regarding complicated features of bacteria released from human individuals in built environments, which will improve public health in urban communities.

## Material and methods

### Air sampling and assessment of environmental factors

Air sampling with monitoring of environmental factors (temperature, humidity, atmospheric pressure, dust particle numbers) was performed at 8:00–20:00 on May 2, June 1, and July 5, 2016 (regular sampling); air analysis in few walkers was also performed at 5:50–7:50 and 20:15–24:15 on July 17, 2017 (baseline sampling). Samples were collected with air samplers (LV40BW, Shibata Scientific Technology Ltd.) placed approximately 3 m from the wall of the pedestrian walkway and 1.5 m from the floor to approximate pedestrian head, according to previous report [27]. Samples were collected with an air sampler [total *n* = 18; 4,800L/each sample [2h-interval collection (regular sampling) or 1 h-interval collection (baseline sampling): air flow, 40L/min]] in removable filters [PTFE-binding glass fiber filter with 0.3μm of pore size (catalog number: T60A20), Shibata] pointed toward the center of the pedestrian walkway (See Fig 1C), and then these samples were used for determining CFU numbers and OTU features. The other factors (temperature, humidity, atmospheric pressure, dust particle numbers) (total *n* = 54) were also recorded by a dust particle counter (handheld particle counter P311, Air Technology, Tokyo, Japan). The number of walkers passing the monitoring place was also manually measured at each time point by three volunteers, and the data were estimated as count average. Weather data were obtained from the Japan Weather Association (http://www.tenki.jp/).

### DNA extraction and 16S rDNA amplicon sequence

At 2 h intervals (regular sampling) or at 1 h interval (baseline sampling), filters were aseptically removed, placed in extraction buffer (PBS with 0.05% Triton X-100) on site, and vortexed on laboratory, according to a previous report [27]. Also, we simply used new and unused filters as a mock control. Specifically, the filters were aseptically placed in the buffer at inside safety cabinet to prevent the contamination of environmental bacteria, and then vortexed on laboratory in a similar manner. The resulting suspension was then equally divided into two and centrifuged, yielding each of the pellets that were used for total genomic DNA extraction and CFU assay (see below). Extraction was performed using a High Pure PCR template preparation kit (Roche Diagnostics GmbH) according to the manufacturer’s instructions. DNA was eluted in 50 μl of elution buffer supplied with the kit and stored at -20°C until use. As mentioned above, unused two filters were employed as a mock control for compensating regular sampling OTU data (with the filter prepared on May 2) and baseline sampling OTU data (with the filter prepared on July 15), respectively; the OTUs obtained from the mock control were removed from those obtained for each sample. Also, the following laboratory procedures were carefully conducted in a safety cabinet with filtered airflow to prevent cross-contamination. All extracted DNA (except for the control) was confirmed by PCR amplification using universal primers that target bacterial 16S rDNA (27F and 1492R) consisting of a condition with 35 cycles of 95°C (2 min), 45°C (30 s), and 72°C (4 min), plus an additional cycle with a chain elongation (20 min) [36]. Because of the sufficient DNA concentrations, the DNA of samples that were PCR positive was used for subsequent amplicon sequence analysis.

Amplicon sequence analysis of samples was conducted as follows. First, the 16S rDNA amplicon library was generated by PCR amplification with 35 cycles (for regular sampling) or 25 cycles plus enrichment of DNA with magnetic beads (Agencort, Beckman Coulter) using the Ion Plus Fragment Library Kit (Life Technologies). Amplicons were sequenced by the Ion S5 and Ion S5 XL Systems (Thermo Fisher). Amplicon libraries were sequenced on a 318 chip using the Ion Torrent Personal Genome Machine (PGM) system and the Ion PGM Hi-Q kit (Life Technologies). Raw reads were processed by the PGM software to remove low quality and polyclonal sequences. 16 rRNA sequences were analyzed using Metagenomics 16S w1.1 ver.5.2 with the Torrent Suite Software (Life Technologies) to perform OTU clustering. OTU annotation was based on applying the Basic Local Alignment Search Tool (BLAST) to data available in the Greengenes database with a baseline of >90% coverage. The analysis, including quality filtering, OTU production, identification of microorganisms, determination of their abundance in the sample and phylogeny generation, was conducted using the QUIIME softwar.

### Bacterial culture

Airborne bacteria derived from the built environments and the walkers themselves were cultivated using SCD (soybean-casein diseated) (Nissui) (nutrient rich medium for walkers) and R2A plates (BD) (nutrient poor medium for built environment).

### Clustering and phylogenic analysis

Cluster analysis was performed using Cluster 3.0 for Mac OS X (Clustering Library 1.52). Phylogenetic trees generated from aligned population structures were constructed and then visualized in Java TreeViewX (version 0.5.0). Specifically, as mentioned above, the OTUs obtained from the mock control were removed from those obtained for each sample. After that the OTU data were treated into a software, ‘Cluster 3.0’, Specifically, the OTU data were transformed to log scales, and then processed with a setting [>80% filtering and Pearson correlation (centered)]. Meanwhile, all data (temperature, humidity, particle number, atmospheric pressure, OUTs, CFUs, walker’s numbers) used for total cluster analysis were similarly converted to an equivalent range from ‘0’ to ‘1’, and then processed with a setting (>80% filtering and Spearman rank correlation). These data were then replaced to the files with a ‘cdt’ extension in the software of Cluster 3.0 for visualizing on the TreeViewX. In addition, the phylum contents of OTUs for mock controls obtained at May 5 and July 15 was shown as S1 Fig. As shown in the supplementary figure, the composition of phylum was very similar with a high correlation coefficient value (*r* = 8.819, *p*<0.001), indicating a baseline as a mock control.

### Statistical analysis

Comparison of the values between factors was conducted using a Mann-Whitney U test. Correlations among factors (walker’s occupancy, temperature, humidity, atmospheric pressure, dust particle numbers, CFUs) were identified by Pearson’s correlation coefficient test. A correlation coefficient value of >0.5 or <−0.05 with a *p*-value < 0.05 was considered significant. All calculations were conducted using Excel for Mac (2001) with Statcel3C.

#### Ethics

The study reported in this manuscript did not involve any human participants, human data, human tissue, data pertaining to specific individuals, or animal experiments. Meanwhile, sampling location and times were selected based on guidance from the City of Sapporo, which maintains the pedestrian space.

## Supporting information

S1 Fig Phylum contents of mock control OTUs obtained at May 5 (S019_HY_9_v1) and July 15 (S005_HY_NC_v1). The composition of phylum was very similar with a high correlation coefficient value (*r* = 8.819, *p*<0.001), indicating a baseline as a mock control.
(TIFF)
