# Supplementary figures and images for: Walker occupancy has an impact on changing airborne bacterial communities in an underground pedestrian space, as small-dust particles increased with raising both temperature and humidity
Source: PLoS One. 2017 Sep 18;12(9):e0184980. doi: 10.1371/journal.pone.0184980 (PMC5602640; doi:10.1371/journal.pone.0184980)

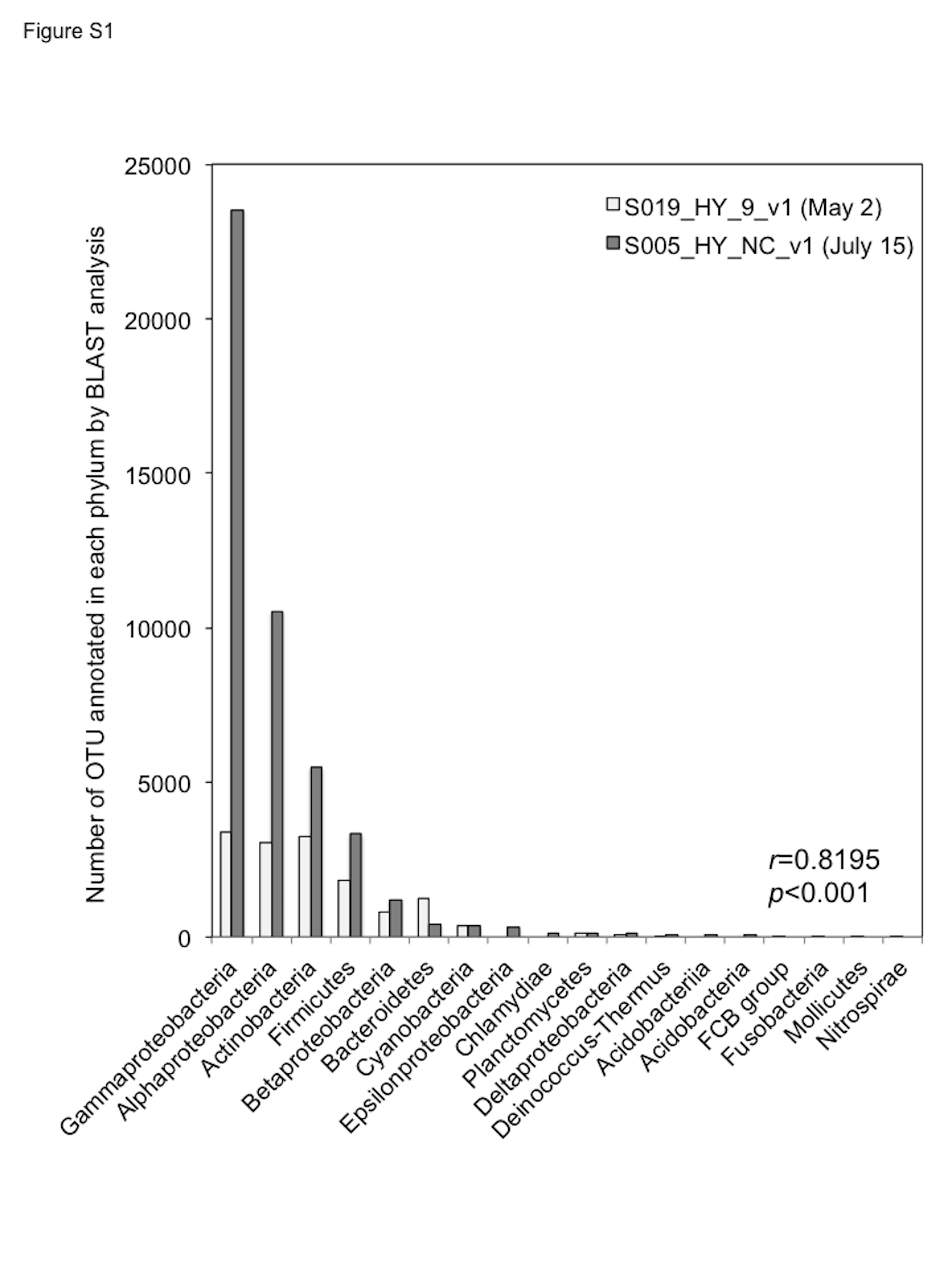

Supplement: S1 Fig — The composition of phylum was very similar with a high correlation coefficient value (r = 8.819, p<0.001), indicating a baseline as a mock control. (TIFF) [file pone.0184980.s001.tiff]
